# Supplementary material for: Diversity and antimicrobial activity of endophytic fungi isolated from Securinega suffruticosa in the Yellow River Delta
Source: PLoS One. 2020 Mar 10;15(3):e0229589. doi: 10.1371/journal.pone.0229589 (PMC7064225; doi:10.1371/journal.pone.0229589)
Supplement: S1 Table — (DOC) [file pone.0229589.s002.doc]

**S1 Table. The accession number of endophytic fungi.**

| Strain No. | accession number |
| --- | --- |
| G1 | MH383162 |
| G2 | MH383163 |
| G3 | MH383164 |
| G4 | MH383165 |
| G5 | MH383166 |
| G6 | MH383167 |
| G7 | MH383168 |
| G8 | MH383169 |
| G9 | MH383170 |
| G10 | MH383171 |
| G11 | MH383172 |
| G12 | MH383173 |
| G13 | MH383174 |
| G14 | MH383175 |
| Y1 | MH383176 |
| Y2 | MH383177 |
| Y3 | MH383178 |
| Y4 | MH383179 |
| Y5 | MH383180 |
| Y6 | MH383181 |
| Y7 | MH383182 |
| Y8 | MH383183 |
| Y9 | MH383184 |
| B5 | MH383185 |
| C1 | MH383186 |
| C2 | MH383187 |
| C3 | MH383188 |
| M1 | MH383189 |
| A1 | MH383190 |
| A2 | MH383191 |
| A3 | MH383192 |
| A4 | MH383193 |
| L1 | MH383194 |
| S1 | MH383195 |
| D1 | MH383196 |
| D2 | MH383197 |
| D3 | MH383198 |
| D4 | MH383199 |
| D5 | MH383200 |
| D6 | MH383201 |
| E1 | MH383202 |
| F1 | MH383203 |
| J1 | MH383204 |
| W1 | MH383205 |
| X1 | MH383206 |
| Z1 | MH383207 |
| H1 | MH383208 |
| T6 | MH383209 |
| P6 | MH383210 |
| K2 | MH383211 |
| K3 | MH383212 |
| N1 | MH383213 |
| N2 | MH383214 |
| N3 | MH383215 |
| N4 | MH383216 |
| N5 | MH383217 |
| N6 | MH383218 |
