# Supplementary material for: Diversity and antimicrobial activity of endophytic fungi isolated from Securinega suffruticosa in the Yellow River Delta
Source: PLoS One. 2020 Mar 10;15(3):e0229589. doi: 10.1371/journal.pone.0229589 (PMC7064225; doi:10.1371/journal.pone.0229589)
Supplement: S2 Table — (DOC) [file pone.0229589.s003.doc]

**S Table. Isolation rate of endophytic fungi from roots, stems and leaves of *S. suffruticosa* species or varieties.**

| *S. suffruticosa* | Roots (segments of tissue, isolation rate) | Stems (segments of tissue, isolation rate) | Leaves (segments of tissue, isolation rate) | Total (segments of tissue, isolation rate) |
| --- | --- | --- | --- | --- |
|  | 170（297,57.24%） | 143（300,47.67%） | 107（348,30.75%） | 420（945,44.44%） |
