# Supplementary material for: Diversity and antimicrobial activity of endophytic fungi isolated from Securinega suffruticosa in the Yellow River Delta
Source: PLoS One. 2020 Mar 10;15(3):e0229589. doi: 10.1371/journal.pone.0229589 (PMC7064225; doi:10.1371/journal.pone.0229589)
Supplement: S1 File — (PDF) [file pone.0229589.s004.pdf]

>G1 [organism=*Chaetomium globosum*] [strain=G1]

ATTACAGAGTTGCAAAACTCCCTAAACCATTGTGAACGTTACCTATACCGT

TGCTTCGGCGGGCGGCCCCGGGGTTTACCCCCCGGGCGCCCCTGGGCCCC

ACCGCGGGCGCCCGCCGGAGGTCACCAAACTCTTGATAATTTATGGCCTC

TCTGAGTCTTCTGTACTGAATAAGTCAAAACTTTCAACAACGGATCTCTTG

GTTCTGGCATCGATGAAGAACGCAGCGAAATGCGATAAGTAATGTGAATT

GCAGAATTCAGTGAATCATCGAATCTTTGAACGCACATTGCGCCCGCCAG

CATTCTGGCGGGCATGCCTGTTCGAGCGTCATTTCAACCATCAAGCCCCCG

GGCTTGTGTTGGGGACCTGCGGCTGCCGCAGGCCCTGAAAAGCAGTGGCG

GGCTCGCTGTCGCACCGAGCGTAGTAGCATACATCTCGCTCTGGTCGCGC

CGCGGGTTCCGGCCGTTAAACCACCTTTTAACCCAAGGTTGACCTCGGATC

AGGTAGGAAGACCCGCTGAATCTA

>G2 [organism=*Chaetomium* sp.] [strain=G2]

TGGAAGTAAAAAAAGCGTAACAAGGTCTCCGTTGGTGAACCAGCGGAGG

GATCATTACAGAGTTGCAAAACTCCCTAAACCATTGTGAACGTTACCTAA

ACCGTTGCTTCGGCGGGCGGCCCCGGGGTTTACCCCCCGGGCGCCCCTGG

GCCCCACCGCGGGCGCCCGCCGGAGGTCACCAAACTCTTGATAATTTATG

GCCTCTCTGAGTCTTCTGTACTGAATAAGTCAAAACTTTCAACAACGGATC

TCTTGGTTCTGGCATCGATGAAGAACGCAGCGAAATGCGATAAGTAATGT

GAATTGCAGAATTCAGTGAATCATCGAATCTTTGAACGCACATTGCGCCC

GCCAGTATTCTGGCGGGCATGCCTGTTCGAGCGTCATTTCAACCATCAAGC

CCCCGGGCTTGTGTTGGGGACCTGCGGCTGCCGCAGGCCCTGAAAAGCAG

TGGCGGGCTCGCTGTCACACCGAGCGTAGTAGCATATATCTCGCTCTGGG

CGTGCTGCGGGTTCCGGCCGTTAAACCACCTTTTAACCCAAGGTTGACCTC

GGATCAGGTAGGAAGACCCGCTGAACTTAAGCATATCAAAAGCCGGGAG

GAA

>G3 [organism=*Chaetomium* sp.] [strain=G3]

TGGAAGGTAAAAAAACGTAACAAGGTCTCCGTTGGTGAACCAGCGGAGG

GATCATTACAGAGTTGCAAAACTCCCTAAACCATTGTGAACGTTACCTAA

ACCGTTGCTTCGGCGGGCGGCGCCGGGGTTTACCCCCCGGGCGCCCCTGG

GCCCCACCGCGGGCGCCCGCCGGAGGTCACCAAACTCTTGATAATTTATG

GCCTCTCTGAGTCTTCTGTACTGAATAAGTCAAAACTTTCAACAACGGATC

TCTTGGTTCTGGCATCGATGAAGAACGCAGCGAAATGCGATAAGTAATGT

GAATTGCAGAATTCAGTGAATCATCGAATCTTTGAACGCACATTGCGCCC

GCCAGTATTCTGGCGGGCATGCCTGTTCGAGCGTCATTTCAACCATCAAGC

CCCGGGCTTGTGTTGGGGACCTGCGGCTGCCGCAGGCCCTGAAAAGCAGT

GGCGGGCTCGCTGTCACACCGAGCGTAGTAGCATACATCTCGCTCTGGGC

GTGCTGCGGGTTCCGGCCGTTAAACCACCTTTTAACCCAAGGTTGACCTCG

GATCAGGTAGGAAGACCCGCTGAACTTAAGCATATCAATAAGCGAAGGA

A

>G4 [organism= *Chaetomium aureum*] [strain=G4]

TGGAAGTAAAAGTCGTAACAAGGTCTCCGTTGGTGAACCAGCGGAGGGAT

CATTACAGAGTTGCAAAACTCCCTAAACCATTGTGGACGCTACCTTTTAAC

GTTGCTTCGGCGGGCGGCCCGCTCCCCTGGAAAGCCCCTGTGGCCGCCCG

GGGCTGCGAGCCCCCCGGCCCCCCTCGCGGGGGCGCCCGCCGGAGGATAC

CCAACTCTTGATTATTTTAGGCCTCTCTGAGTCTTCTGTACTGAATAAGTC

AAAACTTTCAACAACGGATCTCTTGGTTCTGGCATCGATGAAGAACGCAG

CGAAATGCGATAAGTAATGTGAATTGCAGAATTCAGTGAATCATCGAATC

TTTGAACGCACATTGCGCCCGCCAGTATTCTGGCGGGCATGCCTGTTCGAG

CGTCATTTCAACCATCAAGCCCCAGGCTTGTGTTGGGGACCTGCGGCTGCC

GCAGGCCCTGAAATCCAGTGGCGGGTTCGCTGTCACCCCGAGCGTAGTAG

CAATATCTCGCTCAGGGCGTGCTGCGGGCACCGGCCGTTAAAAGCTGCCT

TCTGGCAACACCCAAGGTTGACCTCGGATCAGGTAGGAAGACCCGCTGAA

CTTAAGCATATCAATAAGCGGAGGAA

>G5 [organism=*Chaetomium* sp.] [strain=G5]

TGGGCTCGACTGCAACTCCCTAACCATTGTGACGTTACCTAAACCGTTGCT

TCGGCGGGCGGCCCCGGGGTTTACCCCCCGGGCGCCCCTGGGCCCCACCG

CGGGCGCCCGCCGGAGGTCACCAAACTCTTGATAATTTATGGCCTCTCTG

AGTCTTCTGTACTGAATAAGTCAAAACTTTCAACAACGGATCTCTTGGTTC

TGGCATCGATGAAGAACGCAGCGAAATGCGATAAGTAATGTGAATTGCA

GAATTCAGTGAATCATCGAATCTTTGAACGCACATTGCGCCCGCCAGTATT

CTGGCGGGCATGCCTGTTCGAGCGTCATTTCAACCATCAAGCCCCCGGGC

TTGTGTTGGGGACCTGCGGCTGCCGCAGGCCCTGAAAAGCAGTGGCGGGC

TCGCTGTCACACCGAGCGTAGTAGCATACATCTCGCTCTGGGCGTGCTGC

GGGTTCCGGCCGTTAAACCACCTTTTAACCCAAGGTTGACCTCGGATCAG

GTAGGAAGACCCGCTGAACTTAAGCATATCAAAA

>G6 [organism=*Chaetomium murorum*] [strain=G6]

TTGGAAATGAAAAAAACGTAACAAGGTCTCCGTTGGTGAACCAGCGGAG

GGATCATTACAGAGTTGCAAAACTCCCAAACCATTGTGAACGTTACCTTC

AAACCGTTGCTTCGGCGGGCGGCCCGGGTCCGCCCGGTGCCCCCTGGCCC

CCTCGCGGGGCGCCCGCCGGAGGAAACCCAACTCTTGATACATTATGGCC

TCTCTGAGTCTTCTGTACTGAATAAGTCAAAACTTTCAACAACGGATCTCT

TGGTTCTGGCATCGATGAAGAACGCAGCGAAATGCGATAAGTAATGTGAA

TTGCAGAATTCAGTGAATCATCGAATCTTTGAACGCACATTGCGCCCGCC

AGTATTCTGGCGGGCATGCCTGTTCGAGCGTCATTTCAACCATCAAGCCCC

AGGCTTGTGTTGGGGACCTGCGGCTGCCGCAGGCCCTGAAAACCAGTGGC

GGGCTCGCTGTCACACCGGGCGTAGTAGATTTTATCTCGCTCAGGGCGTG

CTGCGGGTTCCGGCCGTTAAAAAGCCTTTTTTACCCAAGGTTGACCTCGGA

TCAGGTAGGAAGACCCGCTGAACTTAAGCATACAAAGGCGGAAAGGAAT

G

>G7 [organism=*Chaetomium* sp.] [strain=G7]

TGGAAGGTAAAAAAACGTAACAAGGTCTCCGTTGGTGAACCAGCGGAGG

GATCATTACAGAGTTGCAAAACTCCCTAAACCATTGTGAACGTTACCTAA

ACCGTTGCTTCGGCGGGCGGCGCCGGGGTTTACCCCCCGGGCGCCCCTGG

GCCCCACCGCGGGCGCCCGCCGGAGGTCACCAAACTCTTGATAATTTATG

GCCTCTCTGAGTCTTCTGTACTGAATAAGTCAAAACTTTCAACAACGGATC

TCTTGGTTCTGGCATCGATGAAGAACGCAGCGAAATGCGATAAGTAATGT

GAATTGCAGAATTCAGTGAATCATCGAATCTTTGAACGCACATTGCGCCC

GCCAGTATTCTGGCGGGCATGCCTGTTCGAGCGTCATTTCAACCATCAAGC

CCCGGGCTTGTGTTGGGGACCTGCGGCTGCCGCAGGCCCTGAAAAGCAGT

GGCGGGCTCGCTGTCACACCGAGCGTAGTAGCATACATCTCGCTCTGGGC

GTGCTGCGGGTTCCGGCCGTTAAACCACCTTTTAACCCAAGGTTGACCTCG

GATCAGGTAGGAAGACCCGCTGAACTTAAGCATATCAATAAGCGAAGGA

A

>G8 [organism=*Chaetomium* sp.] [strain=G8]

TCCGTAACAAGGTCTCCGTTGGTGAACCAGCGGAGGGATCATTACAGAGT

TGCAAAACTCCCAAACCATTGTGAACGTTACCCATCCCGTTGCTTCGGCGG

GCGGCCCGGGCCCCGTGCCCGGCGCCCCCGGCCCCTCGCGGGCGCCCGC

CGGAGGTAAACCAAACTCTTGAATTGTATGGCCTCTCTGAGTCTTCTGTAC

TGAATAAGTCAAAACTTTCAACAACGGATCTCTTGGTTCTGGCATCGATG

AAGAACGCAGCGAAATGCGATAAGTAATGTGAATTGCAGAATTCAGTGA

ATCATCGAATCTTTGAACGCACATTGCGCCCGCCAGTATTCTGGCGGGCAT

GCCTGTTCGAGCGTCATTTCAACCATCAAGCCCCCGGCTTGTGTTGGGGAC

CTGCGGCTGCCGCAGGCCCTGAAAACCAGTGGCGGGCTCGCTAGTCACTC

CGAGCGTAGTAGTTACATCTCGCTCAGGGCGTGCTGCGGGTTCCGGCCGT

TAAAAAGCCTTATTTACCCCAAGGTTGACCTCGGATCAGGTAGAGAGA

>G9 [organism=*Chaetomium globosum*] [strain=G9]

ATTACAGAGTTGCAAAACTCCCTAAACCATTGTGAACGTTACCTATACCGT

TGCTTCGGCGGGCGGCCCCGGGGTTTACCCCCCGGGCGCCCCTGGGCCCC

ACCGCGGGCGCCCGCCGGAGGTCACCAAACTCTTGATAATTTATGGCCTC

TCTGAGTCTTCTGTACTGAATAAGTCAAAACTTTCAACAACGGATCTCTTG

GTTCTGGCATCGATGAAGAACGCAGCGAAATGCGATAAGTAATGTGAATT

GCAGAATTCAGTGAATCATCGAATCTTTGAACGCACATTGCGCCCGCCAG

CATTCTGGCGGGCATGCCTGTTCGAGCGTCATTTCAACCATCAAGCCCCCG

GGCTTGTGTTGGGGACCTGCGGCTGCCGCAGGCCCTGAAAAGCAGTGGCG

GGCTCGCTGTCGCACCGAGCGTAGTAGCATACATCTCGCTCTGGTCGCGC

CGCGGGTTCCGGCCGTTAAACCACCTTTTAACCCAAGGTTGACCTCGGATC

AGGTAGGAAGACCCGCTGAATCTA

>G10 [organism=*Chaetomium* sp.] [strain=G10]

TGGAAGTAAAAAAAGCGTAACAAGGTCTCCGTTGGTGAACCAGCGGAGG

GATCATTACAGAGTTGCAAAACTCCCTAAACCATTGTGAACGTTACCTAA

ACCGTTGCTTCGGCGGGCGGCCCCGGGGTTTACCCCCCGGGCGCCCCTGG

GCCCCACCGCGGGCGCCCGCCGGAGGTCACCAAACTCTTGATAATTTATG

GCCTCTCTGAGTCTTCTGTACTGAATAAGTCAAAACTTTCAACAACGGATC

TCTTGGTTCTGGCATCGATGAAGAACGCAGCGAAATGCGATAAGTAATGT

GAATTGCAGAATTCAGTGAATCATCGAATCTTTGAACGCACATTGCGCCC

GCCAGTATTCTGGCGGGCATGCCTGTTCGAGCGTCATTTCAACCATCAAGC

CCCCGGGCTTGTGTTGGGGACCTGCGGCTGCCGCAGGCCCTGAAAAGCAG

TGGCGGGCTCGCTGTCACACCGAGCGTAGTAGCATATATCTCGCTCTGGG

CGTGCTGCGGGTTCCGGCCGTTAAACCACCTTTTAACCCAAGGTTGACCTC

GGATCAGGTAGGAAGACCCGCTGAACTTAAGCATATCAAAAGCCGGGAG

GAA

>G11 [organism=*Chaetomium* sp.] [strain=G11]

TGGAAGGTAAAAAAACGTAACAAGGTCTCCGTTGGTGAACCAGCGGAGG

GATCATTACAGAGTTGCAAAACTCCCTAAACCATTGTGAACGTTACCTAA

ACCGTTGCTTCGGCGGGCGGCGCCGGGGTTTACCCCCCGGGCGCCCCTGG

GCCCCACCGCGGGCGCCCGCCGGAGGTCACCAAACTCTTGATAATTTATG

GCCTCTCTGAGTCTTCTGTACTGAATAAGTCAAAACTTTCAACAACGGATC

TCTTGGTTCTGGCATCGATGAAGAACGCAGCGAAATGCGATAAGTAATGT

GAATTGCAGAATTCAGTGAATCATCGAATCTTTGAACGCACATTGCGCCC

GCCAGTATTCTGGCGGGCATGCCTGTTCGAGCGTCATTTCAACCATCAAGC

CCCGGGCTTGTGTTGGGGACCTGCGGCTGCCGCAGGCCCTGAAAAGCAGT

GGCGGGCTCGCTGTCACACCGAGCGTAGTAGCATACATCTCGCTCTGGGC

GTGCTGCGGGTTCCGGCCGTTAAACCACCTTTTAACCCAAGGTTGACCTCG

GATCAGGTAGGAAGACCCGCTGAACTTAAGCATATCAATAAGCGAAGGA

A

>G12 [organism=*Chaetomium murorum*] [strain=G12]

TTGGAAATGAAAAAAACGTAACAAGGTCTCCGTTGGTGAACCAGCGGAG

GGATCATTACAGAGTTGCAAAACTCCCAAACCATTGTGAACGTTACCTTC

AAACCGTTGCTTCGGCGGGCGGCCCGGGTCCGCCCGGTGCCCCCTGGCCC

CCTCGCGGGGCGCCCGCCGGAGGAAACCCAACTCTTGATACATTATGGCC

TCTCTGAGTCTTCTGTACTGAATAAGTCAAAACTTTCAACAACGGATCTCT

TGGTTCTGGCATCGATGAAGAACGCAGCGAAATGCGATAAGTAATGTGAA

TTGCAGAATTCAGTGAATCATCGAATCTTTGAACGCACATTGCGCCCGCC

AGTATTCTGGCGGGCATGCCTGTTCGAGCGTCATTTCAACCATCAAGCCCC

AGGCTTGTGTTGGGGACCTGCGGCTGCCGCAGGCCCTGAAAACCAGTGGC

GGGCTCGCTGTCACACCGGGCGTAGTAGATTTATCTCGCTCAGGGCGTG

CTGCGGGTTCCGGCCGTTAAAAAGCCTTTTTACCCAAGGTTGACCTCGGA

TCAGGTAGGAAGACCCGCTGAACTTAAGCATACAAAGGCGGAAAGGAAT

G

>G13 [organism=*Chaetomium* sp.] [strain=G13]

TGGAAGGTAAAAAAACGTAACAAGGTCTCCGTTGGTGAACCAGCGGAGG

GATCATTACAGAGTTGCAAAACTCCCTAAACCATTGTGAACGTTACCTAA

ACCGTTGCTTCGGCGGGCGGCGCCGGGGTTTACCCCCCGGGCGCCCCTGG

GCCCCACCGCGGGCGCCCGCCGGAGGTCACCAAACTCTTGATAATTTATG

GCCTCTCTGAGTCTTCTGTACTGAATAAGTCAAAACTTTCAACAACGGATC

TCTTGGTTCTGGCATCGATGAAGAACGCAGCGAAATGCGATAAGTAATGT

GAATTGCAGAATTCAGTGAATCATCGAATCTTTGAACGCACATTGCGCCC

GCCAGTATTCTGGCGGGCATGCCTGTTCGAGCGTCATTTCAACCATCAAGC

CCCGGGCTTGTGTTGGGGACCTGCGGCTGCCGCAGGCCCTGAAAAGCAGT

GGCGGGCTCGCTGTCACACCGAGCGTAGTAGCATACATCTCGCTCTGGGC

GTGCTGCGGGTTCCGGCCGTTAAACCACCTTTTAACCCAAGGTTGACCTCG

GATCAGGTAGGAAGACCCGCTGAACTTAAGCATATCAATAAGCGAAGGA

A

>G14 [organism=*Chaetomium* sp.] [strain=G14]

TGGAAGGTAAAAAAACGTAACAAGGTCTCCGTTGGTGAACCAGCGGAGG

GATCATTACAGAGTTGCAAAACTCCCTAAACCATTGTGAACGTTACCTAA

ACCGTTGCTTCGGCGGGCGGCGCCGGGGTTACCCCCCGGGCGCCCCTGG

GCCCCACCGCGGGCGCCCGCCGGAGGTCACCAAACTCTTGATAATTTATG

GCCTCTCTGAGTCTTCTGTACTGAATAAGTCAAAACTTTCAACAACGGATC

TCTTGGTTCTGGCATCGATGAAGAACGCAGCGAAATGCGATAAGTAATGT

GAATTGCAGAATTCAGTGAATCATCGAATCTTTGAACGCACATTGCGCCC

GCCAGTATTCTGGCGGGCATGCCTGTTCGAGCGTCATTTCAACCATCAAGC

CCCGGGCTTGTGTTGGGGACCTGCGGCTGCCGCAGGCCCTGAAAAGCAGT

GGCGGGCTCGCTGTCACACCGAGCGTAGTAGCATACATCTCGCTCTGGGC

GTGCTGCGGGTTCCGGCCGTTAAACCACCTTTTAACCCAAGGTTGACCTCG

GATCAGGTAGGAAGACCCGCTGAACTTAAGCATATCAATAAGCGAAGGA

A

>Y1 [organism=*Fusarium* sp.] [strain=Y1]

GGGGGTTCGGGCTACACTCATCACCCTGTGACATACCTATAACGTTGCCTC

GGCGGGAACAGACGGCCCCGTAACACGGGCCGCCCCCGCCAGAGGACCC

CCTAACTCTGTTTCTATAATGTTTCTTCTGAGTAAACAAGCAAATAAATTA

AAACTTTCAACAACGGATCTCTTGGCTCTGGCATCGATGAAGAACGCAGC

GAAATGCGATAAGTAATGTGAATTGCAGAATTCAGTGAATCATCGAATCT

TTGAACGCACATTGCGCCCGCCAGTATTCTGGCGGGCATGCCTGTTCGAG

CGTCATTACAACCCTCAGGCCCCCGGGCCTGGCGTTGGGGATCGGCGGAA

GCCCCCTGCGGGCACAACGCCGTCCCCCAAATACAGTGGCGGTCCCGCCG

CAGCTTCCATTGCGTAGTAGCTAACACCTCGCAACTGGAGAGCGGCGCGG

CCACGCCGTAAAACACCCAACTTCTGAATGTTGACCTCGAATCAGGTAGG

AATACCCGCTGAACTTAAGCATATCAATAAGCGGAGGAA

>Y2 [organism=*Fusarium* sp.] [strain=Y2]

TGGAAGTAAAAAGTCGTAACAAGGTCTCCGTTGGTGAACCAGCGGAGGG

ATCATTACCGAGTTTACAACTCCCAAACCCCTGTGAACATACCTTAATGTT

GCCTCGGCGGATCAGCCCGCGCCCCGTAAAACGGGACGGCCCGCCAGAG

GACCCAAACTCTAATGTTTCTTATTGTAACTTCTGAGTAAAACAAACAAAT

AAATCAAAACTTTCAACAACGGATCTCTTGGTTCTGGCATCGATGAAGAA

CGCAGCAAAATGCGATAAGTAATGTGAATTGCAGAATTCAGTGAATCATC

GAATCTTTGAACGCACATTGCGCCCGCTGGTATTCCGGCGGGCATGCCTGT

TCGAGCGTCATTTCAACCCTCAAGCCCCCGGGTTTGGTGTTGGGGATCGGC

TCTGCCCTTCTGGGCGGTGCCGCCCCCGAAATACATTGGCGGTCTCGCTGC

AGCCTCCATTGCGTAGTAGCTAACACCTCGCAACTGGAACGCGGCGCGGC

CATGCCGTAAAACCCCAACTTCTGAATGTTGACCTCGGATCAGGTAGGAA

TACCCGCTGAACTTAAGCATATCAATAAGGCGGAGAGA

>Y3 [organism=*Fusarium* sp.] [strain=Y3]

CTTGAAGGTGAAATCGTAACAAGGTCTTCCGTTGGTGAACCAGCGGAGGG

ATCATTACCGAGTTATACAACTCATCAACCCTGTGAACATACCTATAACGT

TGCCTCGGCGGGAACAGACGGCCCCGTAACACGGGCCGCCCCCGCCAGA

GGACCCCCTAACTCTGTTTCTATAATGTTTCTTCTGAGTAAACAAGCAAAT

AAATTAAAACTTTCAACAACGGATCTCTTGGCTCTGGCATCGATGAAGAA

CGCAGCGAAATGCGATAAGTAATGTGAATTGCAGAATTCAGTGAATCATC

GAATCTTTGAACGCACATTGCGCCCGCCAGTATTCTGGCGGGCATGCCTGT

TCGAGCGTCATTACAACCCTCAGGCCCCCGGGCCTGGCGTTGGGGATCGG

CGGAAGCCCCCTGCGGGCACAACGCCGTCCCCCAAATACAGTGGCGGTCC

CGCCGCAGCTTCCATTGCGTAGTAGCTAACACCTCGCAACTGGAGAGCGG

CGCGGCCACGCCGTAAAACTACCCAACTTCTGAATGTTGACCTCGAATCA

GGTAGGAATACCCGCTGAACTTAAGCATATCAATAAGCGGAGGAA

>Y4 [organism=*Fusarium* sp.] [strain=Y4]

GGGGGTTCGGGCTACACTCATCACCCTGTGACATACCTATAACGTTGCCTC

GGCGGGAACAGACGGCCCCGTAACACGGGCCGCCCCCGCCAGAGGACCC

CCTAACTCTGTTTCTATAATGTTTCTTCTGAGTAAACAAGCAAATAAATTA

AAACTTTCAACAACGGATCTCTTGGCTCTGGCATCGATGAAGAACGCAGC

GAAATGCGATAAGTAATGTGAATTGCAGAATTCAGTGAATCATCGAATCT

TTGAACGCACATTGCGCCCGCCAGTATTCTGGCGGGCATGCCTGTTCGAG

CGTCATTACAACCCTCAGGCCCCCGGGCCTGGCGTTGGGGATCGGCGGAA

GCCCCCTGCGGGCACAACGCCGTCCCCCAAATACAGTGGCGGTCCCGCCG

CAGCTTCCATTGCGTAGTAGCTAACACCTCGCAACTGGAGAGCGGCGCGG

CCACGCCGTAAAACACCCAACTTCTGAATGTTGACCTCGAATCAGGTAGG

AATACCCGCTGAACTTAAGCATATCAATAAGCGGAGGAA

>Y5 [organism=*Fusarium* sp.] [strain=Y5]

TGGAAGTAAAAAGTCGTAACAAGGTCTCCGTTGGTGAACCAGCGGAGGG

ATCATTACCGAGTTTACAACTCCCAAACCCCTGTGAACATACCTTAATGTT

GCCTCGGCGGATCAGCCCGCGCCCCGTAAAACGGGACGGCCCGCCAGAG

GACCCAAACTCTAATGTTTCTTATTGTAACTTCTGAGTAAAACAAACAAAT

AAATCAAAACTTTCAACAACGGATCTCTTGGTTCTGGCATCGATGAAGAA

CGCAGCAAAATGCGATAAGTAATGTGAATTGCAGAATTCAGTGAATCATC

GAATCTTTGAACGCACATTGCGCCCGCTGGTATTCCGGCGGGCATGCCTGT

TCGAGCGTCATTTCAACCCTCAAGCCCCCGGGTTTGGTGTTGGGGATCGGC

TCTGCCCTTCTGGGCGGTGCCGCCCCCGAAATACATTGGCGGTCTCGCTGC

AGCCTCCATTGCGTAGTAGCTAACACCTCGCAACTGGAACGCGGCGCGGC

CATGCCGTAAAACCCCAACTTCTGAATGTTGACCTCGGATCAGGTAGGAA

TACCCGCTGAACTTAAGCATATCAATAAGGCGGAGAGA

>Y6 [organism=*Fusarium* sp.] [strain=Y6]

GGGGGTTCGGGCTACACTCATCACCCTGTGACATACCTATAACGTTGCCTC

GGCGGGAACAGACGGCCCCGTAACACGGGCCGCCCCCGCCAGAGGACCC

CCTAACTCTGTTTCTATAATGTTTCTTCTGAGTAAACAAGCAAATAAATTA

AAACTTTCAACAACGGATCTCTTGGCTCTGGCATCGATGAAGAACGCAGC

GAAATGCGATAAGTAATGTGAATTGCAGAATTCAGTGAATCATCGAATCT

TTGAACGCACATTGCGCCCGCCAGTATTCTGGCGGGCATGCCTGTTCGAG

CGTCATTACAACCCTCAGGCCCCCGGGCCTGGCGTTGGGGATCGGCGGAA

GCCCCCTGCGGGCACAACGCCGTCCCCCAAATACAGTGGCGGTCCCGCCG

CAGCTTCCATTGCGTAGTAGCTAACACCTCGCAACTGGAGAGCGGCGCGG

CCACGCCGTAAAACACCCAACTTCTGAATGTTGACCTCGAATCAGGTAGG

AATACCCGCTGAACTTAAGCATATCAATAAGCGGAGGAA

>Y7 [organism=*Fusarium* sp.] [strain=Y7]

GGGGGTTCGGGCTACACTCATCACCCTGTGACATACCTATAACGTTGCCTC

GGCGGGAACAGACGGCCCCGTAACACGGGCCGCCCCCGCCAGAGGACCC

CCTAACTCTGTTTCTATAATGTTTCTTCTGAGTAAACAAGCAAATAAATTA

AAACTTTCAACAACGGATCTCTTGGCTCTGGCATCGATGAAGAACGCAGC

GAAATGCGATAAGTAATGTGAATTGCAGAATTCAGTGAATCATCGAATCT

TTGAACGCACATTGCGCCCGCCAGTATTCTGGCGGGCATGCCTGTTCGAG

CGTCATTACAACCCTCAGGCCCCCGGGCCTGGCGTTGGGGATCGGCGGAA

GCCCCCTGCGGGCACAACGCCGTCCCCCAAATACAGTGGCGGTCCCGCCG

CAGCTTCCATTGCGTAGTAGCTAACACCTCGCAACTGGAGAGCGGCGCGG

CCACGCCGTAAAACACCCAACTTCTGAATGTTGACCTCGAATCAGGTAGG

AATACCCGCTGAACTTAAGCATATCAATAAGCGGAGGAA

>Y8 [organism=*Fusarium* sp.] [strain=Y8]

GGGGGTTCGGGCTACACTCATCACCCTGTGACATACCTATAACGTTGCCTC

GGCGGGAACAGACGGCCCCGTAACACGGGCCGCCCCCGCCAGAGGACCC

CCTAACTCTGTTTCTATAATGTTTCTTCTGAGTAAACAAGCAAATAAATTA

AAACTTTCAACAACGGATCTCTTGGCTCTGGCATCGATGAAGAACGCAGC

GAAATGCGATAAGTAATGTGAATTGCAGAATTCAGTGAATCATCGAATCT

TTGAACGCACATTGCGCCCGCCAGTATTCTGGCGGGCATGCCTGTTCGAG

CGTCATTACAACCCTCAGGCCCCCGGGCCTGGCGTTGGGGATCGGCGGAA

GCCCCCTGCGGGCACAACGCCGTCCCCCAAATACAGTGGCGGTCCCGCCG

CAGCTTCCATTGCGTAGTAGCTAACACCTCGCAACTGGAGAGCGGCGCGG

CCACGCCGTAAAACACCCAACTTCTGAATGTTGACCTCGAATCAGGTAGG

AATACCCGCTGAACTTAAGCATATCAATAAGCGGAGGAA

>Y9 [organism=*Fusarium* sp.] [strain=Y9]

TGGAAGTAAAAAGTCGTAACAAGGTCTCCGTTGGTGAACCAGCGGAGGG

ATCATTACCGAGTTTACAACTCCCAAACCCCTGTGAACATACCTTAATGTT

GCCTCGGCGGATCAGCCCGCGCCCCGTAAAACGGGACGGCCCGCCAGAG

GACCCAAACTCTAATGTTTCTTATTGTAACTTCTGAGTAAAACAAACAAAT

AAATCAAAACTTTCAACAACGGATCTCTTGGTTCTGGCATCGATGAAGAA

CGCAGCAAAATGCGATAAGTAATGTGAATTGCAGAATTCAGTGAATCATC

GAATCTTTGAACGCACATTGCGCCCGCTGGTATTCCGGCGGGCATGCCTGT

TCGAGCGTCATTTCAACCCTCAAGCCCCCGGGTTTGGTGTTGGGGATCGGC

TCTGCCCTTCTGGGCGGTGCCGCCCCCGAAATACATTGGCGGTCTCGCTGC

AGCCTCCATTGCGTAGTAGCTAACACCTCGCAACTGGAACGCGGCGCGGC

CATGCCGTAAAACCCCAACTTCTGAATGTTGACCTCGGATCAGGTAGGAA

TACCCGCTGAACTTAAGCATATCAATAAGGCGGAGAGA

>B5 [organism=*Trichoderma harzianum*] [strain=B5]

ACCGAATTTAAACTCCCAAACCCAATGTGAACGTTACCAAACTGTNGCCT

CGGCGGGATCTCTGCCCCGGGTGCGTCGCAGCCCCGGACCAAGGCGCCCG

CCGGAAGACCAACCTAAAACTCTTATTGTATACCCCCTCGCGGGTTTTTT

TTATAATCTGAGCCTTCTCGGCGCCTCTCGTAGGCGTTTCGAAAATGAATC

AAAACTTTCCAACAACGGAATCTCTTGGTTCTGGCATCGATAAAGAACCC

ACCAAAATGCAATAAATAATGTGAATTGCAGAATTCAGTGAATCATCGAA

TCTTTGAACGCACATTGCGCCCGCCAGTATTCTGGCGGGCATGCCTGTCCG

AGCGTCATTTCAACCCTCGAACCCCTCCGGGGGGTCGGCGTTGGGGATCG

GCCCTCCCTTAGCGGGTGGCCGTCTCCGAAATACAGTGGCGGTCTCGCCG

CAGCCTCTCCTGCGCAGTAGTTTGCACACTCGCATCGGGAGCGCGGCGCG

TCCACAGCCGTTAAACACCCAACTTCTGAAATGTTGACCTCGAGT

>C1 [organism= *Colletotrichum* sp.] [strain=C1]

AGGGATCATTACTGAGTTACCGCTCTATAACCCTTTGTGAACATACCTAAC

CGTTGCTTCGGCGGGCAGGGGAAGCCTCTCGCGGGCCTCCCCTCCCGGCG

CCGGCCCCCACCACGGGGACGGGGCGCCCGCCGGAGGAAACCAAACTCT

ATTTACACGACGTCTCTTCTGAGTGGCACAAGCAAATAATTAAAACTTTTA

ACAACGGATCTCTTGGTTCTGGCATCGATGAAGAACGCAGCGAAATGCGA

TAAGTAATGTGAATTGCAGAATTCAGTGAATCATCGAATCTTTGAACGCA

CATTGCGCTCGCCAGCATTCTGGCGAGCATGCCTGTTCGAGCGTCATTTCA

ACCCTCAAGCACCGCTTGGTTTTGGGGCCCCACGGCACACGTGGGCCCTT

AAAGGTAGTGGCGGACCCTCCCGGAGCCTCCTTTGCGTAGTAACTAACGT

CTCGCACTGGGATCCGGAGGGACTCTTGCCGTAAAACCCCCCAATTCTTTA

CAGGTTGACCTCGGATCAGGTAGGAATACCCGCTGAA

> C2 [organism=*Colletotrichum* sp.] [strain=C2]

AGGGATCATTACTGAGTTTACGCTCTACAACCCTTTGTGAACATACCTATA

ACTGTTGCTTCGGCGGGTAGGGTCTCCGCGACCCTCCCGGCCTCCCGCCCC

CGGGCGGGTCGGCGCCCGCCGGAGGATAACCAAACTCTGATTTAACGACG

TTTCTTCTGAGTGGTACAAGCAAATAATCAAAACTTTTAACAACGGATCTC

TTGGTTCTGGCATCGATGAAGAACGCAGCGAAATGCGATAAGTAATGTGA

ATTGCAGAATTCAGTGAATCATCGAATCTTTGAACGCACATTGCGCCCGC

CAGCATTCTGGCGGGCATGCCTGTTCGAGCGTCATTTCAACCCTCAAGCTC

TGCTTGGTGTTGGGGCCCTACAGCTGATGTAGGCCCTCAAAGGTAGTGGC

GGACCCTCCCGGAGCCTCCTTTGCGTAGTAACTTTACGTCTCGCACTGGGA

TCCGGAGGGACTCTTGCCGTAAAACCCCCAATTTTCCAAAGGTTGACCTC

GGATCAGGTAGGAATACCCGCTGAAC

>C3 [organism= *Colletotrichum* sp.] [strain=C3]

AGGGATCATTACTGAGTTACCGCTCTATAACCCTTTGTGAACATACCTAAC

CGTTGCTTCGGCGGGCAGGGGAAGCCTCTCGCGGGCCTCCCCTCCCGGCG

CCGGCCCCCACCACGGGGACGGGGCGCCCGCCGGAGGAAACCAAACTCT

ATTTACACGACGTCTCTTCTGAGTGGCACAAGCAAATAATTAAAACTTTTA

ACAACGGATCTCTTGGTTCTGGCATCGATGAAGAACGCAGCGAAATGCGA

TAAGTAATGTGAATTGCAGAATTCAGTGAATCATCGAATCTTTGAACGCA

CATTGCGCTCGCCAGCATTCTGGCGAGCATGCCTGTTCGAGCGTCATTTCA

ACCCTCAAGCACCGCTTGGTTTTGGGGCCCCACGGCACACGTGGGCCCTT

AAAGGTAGTGGCGGACCCTCCCGGAGCCTCCTTTGCGTAGTAACTAACGT

CTCGCACTGGGATCCGGAGGGACTCTTGCCGTAAAACCCCCCAATTCTTTA

CAGGTTGACCTCGGATCAGGTAGGAATACCCGCTGAA

>M1 [organism=*Diaporthe* sp.] [strain=M1]

AACAAGGTCTCCGTTGGTGAACCAGCGGAGGGATCATTGCTGGAACGCGC

CCCTGGCGCACCCAGAAACCCTTTGTGAACTTATACCTTACTGTTGCCTCG

GCGCAGGCCGTCCCCTGATGGGGTCCCTCTGGAGACAGAGGAGCAGCCGG

CCGGTGGCCAAATTAACTCTGTTTTTACACTGAAACTCTGAGTACAAAAC

ATAAATGAATCAAAACTTTCAACAACGGATCTCTTGGTTCTGGCATCGAT

GAAGAACGCAGCGAAATGCGATAAGTAATGTGAATTGCAGAATTCAGTG

AATCATCGAATCTTTGAACGCACATTGCGCCCTCTGGTATTCCGGAGGGC

ATGCCTGTTCGAGCGTCATTTCAACCCTCAAGCCTGGCTTGGTGTTGGGGC

ACTGCTCTCTGACGAGAGCAGGCCCTGAAATATAGTGGCGAGCTCGCCAG

GACTCCGAGCGTAGTAGTTAAACCCTCGCTTTGGAAGGCCTGGCGGTGCC

CTGCCGTTAAACCCCAACTTCTGAAAATTTGACCTCGGATCAGGTAGGAA

TACCCGCTGAACTTAAGCATA

>A1 [organism=*Phoma* sp.] [strain=A1]

CATTACAATTTTGGTACATCGAGTCGCCCCTCGGGGCGGCTCCGGTGTCAC

CAATCGAGATACCAACCCATGTCTTTTGAGCACCTCACGTTTCCTCGGCAG

GCTCGCCTGCCAATGGGGACCCCTTTAAAACCTTTTTATGTATGCAGTAAA

CGTCTATAAACAAATGAAATCAAAACTTTCAACAACGGATCTCTTGGTTCT

GGCATCGATGAAGAACGCAGCGAAATGCGATAAGTAGTGTGAATTGCAG

AATTCAGTGAATCATCGAATCTTTGAACGCACATTGCGCCCTTTGGTATTC

CTTAGGGCATGCCTGTTCGAGCGTCATCTAAACCTTCAAGCACTGCTTGGT

GTTGGGCGTCTTTGTCCCGCCTCCTGGCGCGGACTCGCCTTAAAATCATTG

GCAGCCTGTGTATTGGCTTCGAGCGCAGCAGACTCGCGCTTTCGTCTCCTT

TAGCACAGGCGTCCAGCAAGCCTAACCCACTAAGTTTGACCTCGGATCAG

GTAGGGATACCCGCTGAACTTAAGCAACAA

>A2 [organism=*Phomoa* sp.] [strain=A2]

AAGTCGTAACAAGGTTTCCGTAGGTGAACCTGCGGAAGGATCATTACCTA

GAGTTGTAGGCTTTGCCTGCTATCTCTTACCCATGTCTTTTAAGTACCTTCG

TTTCCTCGGCGGGTTCGCCCGCCGATTGGACAATTTAAACCATTTGCAGTT

GCAATCAGCGTCTGAAAAAACTTAATAGTTACAACTTTCAACAACGGATC

TCTTGGTTCTGGCATCGATGAAGAACGCAGCGAAATGCGATAAGTAGTGT

GAATTGCAGAATTCAGTGAATCATCGAATCTTTGAACGCACATTGCGCCC

CTTGGTATTCCATGGGGCATGCCTGTTCGAGCGTCATTTGTACCTTCAAGC

TCTGCTTGGTGTTGGGTGTTTGTCTCGCCTCTGCGTGTAGACTCGCCTCAA

AACAATTGGCAGCCGGCGTATTGATTTCGGAGCGCAGTACATCTCGCGCT

TTGCACTCATAACGACGACGTCCAAAAGTACATTTTTACACTCTTGACCTC

GGATCAGGTAGGGATACCCGCTGAACTTAAGCATATT

>A3 [organism= *Phoma multirostrata*] [strain= A3]

AAACCTGCGGAAGGATCATTACCTAGAGTTGTAGGCTTTGCCTGCTATCTC

TTACCCATGTCTTTTGAGTACCTTCGTTTCCTCGGCGGGTCCGCCCGCCGA

TTGGACACATTTAAACCCTTTGTAGTTGCAATCAGCGTCTGAAAAACTTTA

ATAGTTACAACTTTCAACAACGGATCTCTTGGTTCTGGCATCGATGAAGA

ACGCAGCGAAATGCGATAAGTAGTGTGAATTGCAGAATTCAGTGAATCAT

CGAATCTTTGAACGCACATTGCGCCCCTTGGTATTCCATGGGGCATGCCTG

TTCGAGCGTCATTTGTACCTTCAAGCTTTGCTTGGTGTTGGGTGTTTGTCTC

GCCTCTGCGCGCAGACTCGCCTCAAAACAATTGGCAGCCGGCGTATTGAT

TTCGGAGCGCAGTACATCTCGCGCTTTGCACTCATAACGACGACGTCCAA

AAAGTACATTTCTTACACTCTTGACCTCGGATCAGGTAGGGATACCCGCTG

AACTTAAGCATATCAATAAGCGGAGGAA

>A4 [organism=*Phoma* sp.] [strain=A4]

CATTACAATTTTGGTACATCGAGTCGCCCCTCGGGGCGGCTCCGGTGTCAC

CAATCGAGATACCAACCCATGTCTTTTGAGCACCTCACGTTTCCTCGGCAG

GCTCGCCTGCCAATGGGGACCCCTTTAAAACCTTTTTATGTATGCAGTAAA

CGTCTATAAACAAATGAAATCAAAACTTTCAACAACGGATCTCTTGGTTCT

GGCATCGATGAAGAACGCAGCGAAATGCGATAAGTAGTGTGAATTGCAG

AATTCAGTGAATCATCGAATCTTTGAACGCACATTGCGCCCTTTGGTATTC

CTTAGGGCATGCCTGTTCGAGCGTCATCTAAACCTTCAAGCACTGCTTGGT

GTTGGGCGTCTTTGTCCCGCCTCCTGGCGCGGACTCGCCTTAAAATCATTG

GCAGCCTGTGTATTGGCTTCGAGCGCAGCAGACTCGCGCTTTCGTCTCCTT

TAGCACAGGCGTCCAGCAAGCCTAACCCACTAAGTTTGACCTCGGATCAG

GTAGGGATACCCGCTGAACTTAAGCAACAA

>L1 [organism=*Phomopsis* sp.] [strain=L1]

CTCCGTAGGTGAACCTGCGGAGGGATCATTGCTGGAACGCGCCCCAGGCG

CACCCAGAAACCCTTTGTGAACTTATACCTTACTGTTGCCTCGGCGCATGC

CGGCCCCCCTGGGGGCCCCTCCTTCTGGAGGAGCAGGCACGCCGGCGGCC

AACCTAACTCTTGTTTTTACACTGAAACTCTGAGAATAAAACATAAATGA

ATCAAAACTTTCAACAACGGATCTCTTGGTTCTGGCATCGATGAAGAACG

CAGCGAAATGCGATAAGTAATGTGAATTGCAGAATTCAGTGAATCATCGA

ATCTTTGAACGCACATTGCGCCCTCTGGTATTCCGGAGGGCATGCCTGTTC

GAGCGTCATTTCAACCCTCAAGCCTGGCTTGGTGATGGGGCACTGCTTCTT

ACCCAAGAAGCAGGCCCTGAAATTCAGTGGCGAGCTCGCCAGGACCCCG

AGCGCAGTAGTTAAACCCTCGCTCTGGAAGGCCCTGGCGGTGCCCTGCCG

TTAAACCCCCAACTTCTGAAAATTGACCTCGGA

>S1 [organism= *Daldinia* sp.] [strain=S1]

TATCAGCGACGACTCCACCCTTTGTGAAACTTACCGTCGTTGCCTCGGCGG

GCTGCGCTTACCCTGTAGCTACCCTGTAGCTACCCGGTAGGCGCGCTCCAA

GCCCGCCGGTGGACCACTAAACTCTGTTTTAATACTGAATCTCTGAATGCT

TCAACTTAATAAGTTAAAACTTTCAACAACGGATCTCTTGGTTCTGGCATC

GATGAAGAACGCAGCGAAATGCGATAAGTAATGTGAATTCGAGAATTCA

GTGAATCATCGAATCTTTGAACGCACATTGCGCCCATTAGTATTCTAGTGG

GCATGCCTATTCGAGCGTCATTTCAACCCTTAAGCCTTAGTTGCTTAGCGT

TGGGAGTCTGCGCTGTACTTGTTACGGCGCAGTTCCTCAAAGTGATTGGCG

GAGTTAGGGCATACTCTAAGCGTAGTAATATTTCTTCTCGCTTCTGTAGTT

GTCCTGGCGGCTTGCCGTTAAACCCCTATATTTCTAGTGGTTGACCTCGG

ATTAGGCTAGGA

>D1 [organism= *Cladosporium ramotenellum*] [strain=D1]

CAATTGGAAGTAAAAGTCGTAACAAGGTCTCCGTAGGTGAACCTGCGGAG

GGATCATTACAAGTGACCCCGGCTACGGCCGGGATGTTCATAACCCTTTG

TTGTCCGACTCTGTTGCCTCCGGGGCGACCCTGCCTTCGGGCGGGGGCTCC

GGGTGGACACTTCAAACTCTTGCGTAACTTTGCAGTCTGAGTAAACTTAAT

TAATAAATTAAAACTTTTAACAACGGATCTCTTGGTTCTGGCATCGATGAA

GAACGCAGCGAAATGCGATAAGTAATGTGAATTGCAGAATTCAGTGAATC

ATCGAATCTTTGAACGCACATTGCGCCCCCTGGTATTCCGGGGGGCATGC

CTGTTCGAGCGTCATTTCACCACTCAAGCCTCGCTTGGTATTGGGCAACGC

GGTCCGCCGCGTGCCTCAAATCGTCCGGCTGGGTCTTCTGTCCCCTAAGCG

TTGTGGAAACTATTCGCTAAAGGGTGTTCGGGAGGCTACGCCGTAAAACA

ACCCCATTTCTAAGGTTGACCTCGGATCAGGTAGGGATACCCGCTGAACT

TAAGCATATCAATAAGCGGAGGAA

>D2 [organism= *Cladosporium ramotenellum*] [strain=D2]

CAATTGGAAGTAAAAGTCGTAACAAGGTCTCCGTAGGTGAACCTGCGGAG

GGATCATTACAAGTGACCCCGGCTACGGCCGGGATGTTCATAACCCTTTG

TTGTCCGACTCTGTTGCCTCCGGGGCGACCCTGCCTTCGGGCGGGGGCTCC

GGGTGGACACTTCAAACTCTTGCGTAACTTTGCAGTCTGAGTAAACTTAAT

TAATAAATTAAAACTTTTAACAACGGATCTCTTGGTTCTGGCATCGATGAA

GAACGCAGCGAAATGCGATAAGTAATGTGAATTGCAGAATTCAGTGAATC

ATCGAATCTTTGAACGCACATTGCGCCCCCTGGTATTCCGGGGGGCATGC

CTGTTCGAGCGTCATTTCACCACTCAAGCCTCGCTTGGTATTGGGCAACGC

GGTCCGCCGCGTGCCTCAAATCGTCCGGCTGGGTCTTCTGTCCCCTAAGCG

TTGTGGAAACTATTCGCTAAAGGGTGTTCGGGAGGCTACGCCGTAAAACA

ACCCCATTTCTAAGGTTGACCTCGGATCAGGTAGGGATACCCGCTGAACT

TAAGCATATCAATAAGCGGAGGAA

>D3 [organism=*Cladosporium* sp.] [strain=D3]

TTTAGAGGAAGTAAAAGTCGTAACAAGGTCTCCGTAGGTGAACCTGCGGA

GGGATCATTACAAGTTGACCCCGGCCCTCGGGCCGGGATGTTCACAACCC

TTTGTTGTCCGACTCTGTTGCCTCCGGGGCGACCCTGCCTCCGGGCGGGGG

CCCCGGGTGGACATTTCAAACTCTTGCGTAACTTTGCAGTCTGAGTAAATT

TAATTAATAAATTAAAACTTTCAACAACGGATCTCTTGGTTCTGGCATCGA

TGAAGAACGCAGCGAAATGCGATAAGTAATGTGAATTGCAGAATTCAGTG

AATCATCGAATCTTTGAACGCACATTGCGCCCCCTGGTATTCCGGGGGGC

ATGCCTGTTCGAGCGTCATTTCACCACTCAAGCCTCGCTTGGTATTGGGCG

ACGCGGTCCGCCGCGCGCCTCAAATCGACCGGCTGGGTCTTTCGTCCCCTC

AGCGTTGTGGAAACTATTCGCTAAAGGGTGCCGCGGGAGGCCACGCCGTA

AAACAACCCCATTTCTAAGGTTGACCTCGGATCAGGTAGGGATACCCGCT

GAACTTAAGCATATCAATAAGCGGAGGAAA

> D4 [organism=*Cladosporium* sp.] [strain=D4]

TCATTTAGAGGAAGTAAAAGTCGTAACAAGGTCTCCGTAGGTGAACCTGC

GGAGGGATCATTAACCGTAACACAGACTCGGCGCGGACTCCGGTCCCCGA

GGAAATGTCACACCCTTTGTTGTCCGACCACGTTGCCTCGGGGGTGACCCT

GCCTTCGGGCTCGGTCCCCCCGGTGGACCAAACCAAACTCTTGCGTAACTT

TGCAGTCTGAGTGATTTTATAAATAAATCAAAACTTTCAACAACGGATCTC

TTGGTTCTGGCATCGATGAAGAACGCAGCGAAATGCGATAAGTAATGTGA

ATTGCAGAATTCAGTGAATCATCGAATCTTTGAACGCACATTGCGCCCCGT

GGTATTCCGCGGGGCATGCCTGTTCGAGCGTCATTACACCACTCAAGCCTC

GCTTGGTATTGGGCGACGCGGTCCGCCGCGCGCCTCAAATCTTCCGACTG

GGTTCGATCGTCCCTCAGCGTTGTGGAAACTATTCGCTAAAGGTGCACTTT

CGGGTCACGCCGTTAAACAAACCCCATTCAAAGGTTGACCTCGGATCAGG

TAGGGATACCCGCTGAACTTAAGCATATCAATAAGCGGAGGAAT

>D5 [organism= *Cladosporium ramotenellum*] [strain=D5]

CAATTGGAAGTAAAAGTCGTAACAAGGTCTCCGTAGGTGAACCTGCGGAG

GGATCATTACAAGTGACCCCGGCTACGGCCGGGATGTTCATAACCCTTTG

TTGTCCGACTCTGTTGCCTCCGGGGCGACCCTGCCTTCGGGCGGGGGCTCC

GGGTGGACACTTCAAACTCTTGCGTAACTTTGCAGTCTGAGTAAACTTAAT

TAATAAATTAAAACTTTTAACAACGGATCTCTTGGTTCTGGCATCGATGAA

GAACGCAGCGAAATGCGATAAGTAATGTGAATTGCAGAATTCAGTGAATC

ATCGAATCTTTGAACGCACATTGCGCCCCCTGGTATTCCGGGGGGCATGC

CTGTTCGAGCGTCATTTCACCACTCAAGCCTCGCTTGGTATTGGGCAACGC

GGTCCGCCGCGTGCCTCAAATCGTCCGGCTGGGTCTTCTGTCCCCTAAGCG

TTGTGGAAACTATTCGCTAAAGGGTGTTCGGGAGGCTACGCCGTAAAACA

ACCCCATTTCTAAGGTTGACCTCGGATCAGGTAGGGATACCCGCTGAACT

TAAGCATATCAATAAGCGGAGGAA

>D6 [organism= *Cladosporium ramotenellum*] [strain=D6]

CAATTGGAAGTAAAAGTCGTAACAAGGTCTCCGTAGGTGAACCTGCGGAG

GGATCATTACAAGTGACCCCGGCTACGGCCGGGATGTTCATAACCCTTTG

TTGTCCGACTCTGTTGCCTCCGGGGCGACCCTGCCTTCGGGCGGGGGCTCC

GGGTGGACACTTCAAACTCTTGCGTAACTTTGCAGTCTGAGTAAACTTAAT

TAATAAATTAAAACTTTTAACAACGGATCTCTTGGTTCTGGCATCGATGAA

GAACGCAGCGAAATGCGATAAGTAATGTGAATTGCAGAATTCAGTGAATC
ATCGAATCTTTGAACGCACATTGCGCCCCCTGGTATTCCGGGGGGCATGC
CTGTTCGAGCGTCATTTCACCACTCAAGCCTCGCTTGGTATTGGGCAACGC
GGTCCGCCGCGTGCCTCAAATCGTCCGGCTGGGTCTTCTGTCCCCTAAGCG
TTGTGGAAACTATTCGCTAAAGGGTGTTCGGGAGGCTACGCCGTAAAACA
ACCCCATTTCTAAGGTTGACCTCGGATCAGGTAGGGATACCCGCTGAACT
TAAGCATATCAATAAGCGGAGGAA

>E1 [organism=*Alternaria* sp.] [strain= E1]

ATCATTACACAAATATGAAGGCGGGCTGGAATCTCTCGGGGTTACAGCCT
TGCTGAATTATTCACCCTTGTCTTTTGCGTACTTCTTGTTTCCTTGGTGGGT
TCGCCCACCACTAGGACAAACATAAACCTTTTGTAATTGCAATCAGCGTC
AGTAACAAATTAATAATTACAACTTTCAACAACGGATCTCTTGGTTCTGGC
ATCGATGAAGAACGCAGCGAAATGCGATAAGTAGTGTGAATTGCAGAATT
CAGTGAATCATCGAATCTTTGAACGCACATTGCGCCCTTTGGTATTCCAAA
GGGCATGCCTGTTCGAGCGTCATTTGTACCCTCAAGCTTTGCTTGGTGTTG
GGCGTCTTGTCTCTAGCTTTGCTGGAGACTCGCCTTAAAGTAATTGGCAGC
CGGCCTACTGGTTTCGGAGCGCAGCACAAGTCGCACTCTCTATCAGCAAA
GGTCTAGCATCCATTAAGCCTTTTTTCAACTTTTGACCTCGGATCAGGTAG
GGATACC

>F1 [organism= *Peyronellaea* sp.] [strain= F1]

ACATGCTGGCGTCGGTACCTAGAGTTGTAGGCTTTGCCTGCTATCTCTTAC
CCATGTCTTTTAAGTACCTTACGTTTCCTCGGCGGGTCCGCCCGCCGATTG

GACAATTTAAACCATTTGCAGTTGCAATCAGCGTCTGAAAAAACTTAATA

GTTACAACTTTCAACAACGGATCTCTTGGTTCTGGCATCGATGAAGAACG

CAGCGAAATGCGATAAGTAGTGTGAATTGCAGAATTCAGTGAATCATCGA

ATCTTTGAACGCACATTGCGCCCCTTGGTATTCCATGGGGCATGCCTGTTC

GAGCGTCATTTGTACCTTCAAGCTCTGCTTGGTGTTGGGTGTTTGTCTCGC

CTCTGCGTGTAGACTCGCCTCAAAACAATTGGCAGCCGGCGTATTGATTTC

GGAGCGCAGTACATCTCGCGCTTTGCACTCATAACGACGACGTCCAAAAG

TACATTTTTACACTCTTGACCTCGGATCAGGTAGGGATACCCGCTGAACTT

AAGCATATACAAA

>J1 [organism= *Periconia* sp.] [strain= J1]

TAGAGGAAGTAAAAGTCGTAACAAGGTTTCCGTAGGTGAACCTGCGGAA

GGATTTTAGAGGAAGTAAAAGTCGTAACAAGGTTTCCGTAGGTGAACCTG

CGGAAGGATCATTACGAAATTCGGCGCGCTTCGGCGCTGCCTTATTCACC

CACCCTTTGTCTACGTGTACCTCTATAGCTTCCTCGGCGGGCTCGCCCGCC

GCCAGGAACCCACGAAACCCCTTGCATTATACACGAAAACTTCTGATAAC

AAACCTAAATTATTACAACTTTCAACAATGGATCTCTTGGTTCTGGCATCG

ATGAAGAACGCAGCGAAATGCGATAAGTAGTGTGAATTGCAGAATTCAGT

GAATCATCGAATCTTTGAACGCACATTGCGGCCATAGGTATTCCTTTGGCC

ATGCCTGTTCGAGCGTCATTTACACCCTCAAGCATAGCTTGGTGTTGGGCG

TCTGTCCCGCCGTTTTCGCGCGCGGACTCGCCTCAAAGTCATTGGCGGCGG

TCGTGCCGGCTCCTCGCGCAGCACATTTGCGCTTCTCGGAGGCCCGGCGG

ATCCGCGCTCCAGCAAAGACCTTTTATGACTTGACCTCGGATCAGGTAGG

GATACCCGCTGAACTTAAGCATATCAATAAGCGGAGGAC

> W1 [organism= *Curvularia* sp.] [strain= W1]

ATGACGTAATATGAAGGCTGTACGCGGCTGTGCTCTCGGGCCAGTTTTGC

GGAGGCTGAATTATTTATTACCCTTGTCTTTTGCGCACTTGTTGTTTCCTGG

GCGGGTTCGCCCGCCACCAGGACCACATCATAAACCTTTTTTATGCAGTTG

CAATCAGCGTCAGTATAACAAATGTAAATCATTTACAACTTTCAACAACG

GATCTCTTGGTTCTGGCATCGATGAAGAACGCAGCGAAATGCGATACGTA

GTGTGAATTGCAGAATTCAGTGAATCATCGAATCTTTGAACGCACATTGC

GCCCTTTGGTATTCCAAAGGGCATGCCTGTTCGAGCGTCATTTGTACCCTC

AAGCTTTGCTTGGTGTTGGGCGTTTTTTGTCTTTGGTTGCCAAAGACTCGC

CTTAAAAGGATTGGCAGCCGGCCTACTGGTTTCGCAGCGCAGCACATTTTT

GCGCTTGCAATCAGCAAAAGAGGACGGCAATCCATCAAGACTCCTTCTCA

CGTTTGACCTCGGATCAGGTAGGGATACCCGCTGAACTTAAGCATATCAA

TAAGCGGGAGGAAAA

> X1 [organism= *Paraphaeosphaeria* sp.] [strain= X1]

GGGTCCGGTTACGGTGTGGTCGCGGCCTCCAGGGGTCTTCCCCTGGGCGG

TAGAGGTAACACTTTCACGCGCCACATGTCTGAATCCTTTTTTTACGAGCA

CCTTTCGTTCTCCTTCGGCGGGGCAGCCTGCCGTTGGAACCTATCAAAACC

TTTTTTTGCATCTAGCATTACCTGTTCTGATACAAACAATCGTTACAACTTT

CAACAATGGATCTCTTGGCTCTGGCATCGATGAAGAACGCAGCGAAATGC

GATAAGTAGTGTGAATTGCAGAATTCAGTGAATCATCGAATCTTTGAACG

CACATTGCGCCCCTTGGTATTCCATGGGGCATGCCTGTTCGAGCGTCATCT

AAACCCTCAAGCTCTGCTTGGTGTTGGGCGTCTGTCCCGCCTCTGCGCGTG

GACTCGCCCCAAATGCATTGGCAGCAGTCTTTGCCTTCCTCTTGTGCAGTA

CAGTGCGCTTATAGAGGGGGCTAGGCCTGCGTCCATGAAGCAACATTTAT

CAACTTTGACCTCGGATCAGGTAGGGATACCCGCTGAACTTAAGCATATC

AATGGAAAAGC

> Z1 [organism= *Leptosphaeria* sp.] [strain= Z1]

ACCCGTAACAAGGTTTCCGTAAGGTGAACCTGCGGAAGGATCATTACATT

CAGTAGCTTGCTACTGTTAGGGGGGTCCATTAGTCTGTATAGTATCACTAC

TGATGAGCAGCACGACTCCCTGTCTATACCCTTGTCTTTTGCGCACTCATG

TTTCCTCGGCGGCTTGATACCCGCCGGCTGGATCAATCTATAACCTTTTTA

ATTTTCAATCAGCGTCTGAAAAACCTAATAATTACAACTTTCAACAACGG

ATCTCTTGGTTCTGGCATCGATGAAGAACGCAGCGAAATGCGATAAGTAG

TGTGAATTGCAGAATTCAGTGAATCATCGAATCTTTGAACGCACATTGCG

CCCCTTGGTATTCCATGGGGCATGCCTGTTCGAGCGTCATTTGTACCCTCA

AGCTCTGCTTGGTGTTGGGNTGTTTGTCCACTGTCGTGGACTCGCCTTAAA

GTCATTGGCAGCCAGTGTTTTGGTATTGAAGCGCAGCACATTTTGCGCCTC

TAGCCTAGAGCACTCGCGTCCAGTAAGCCTTTTTCCACTTTTGACCTCGGA

TCGGAAGGTAG

>H1 [organism=*Mycosphaerella* sp.] [strain= H1]

AATAATGAATGAGGATCCGGGTCCGACCTCCAACCCTTTTGTGAACCAAC

CTGTTGCTTCGGGGGAGACCCCGCCGTTTCGGCGGCGGCGGCTCCCCCGG

AGGCCCATTAACACTGCGTAACTGTTTAGTCGGAGTCTTAATAAATCAATC

AAAACTTTCAACAACGGATCTCTTGGTTCTGGCATCGATGAAGAACGCAG

CGAAATGCGATAAGTAATGTGAATTGCAGAATTCAGTGAATCATCGAATC

TTTGAACGCACATTGCGCCCCCTGGTATTCCGGGGGGCATGCCTGTCCGA

GCGTCATTACACCCCTCAAGCCTGGCTTGGTATTGGGCGTCGCGGGCCTCG

CCCGCGCGCCTTAAAGTCTCCCCGGCTGGACCGTTTGTCCCTAAAGCGTCG

TGCAAACCTCGCGGTGGGATTTGATGGGCCGGCCGTTAAATCTTTATCAA

AGGTTGACCTCGGATCAGGTAGGGATACCCGCTGAACTTAAGCATATCAA

TAAGCGGGGAAGAG

>T6 [organism=*Rhizopycnis* sp.] [strain=T6]

TTCCGTAGGTGAACCTGCGGAAGGATCATTAACGATTTCGGTTTACACCGT

TCTCTACCTATGTCTACGCGTACCACTTGTTTCCTCGGGGGGCTTGCCCCC

CGTTAGGACTCTTCAATCAACCTTTTTGCAATAGCAGTCAGCGTCTGATAC

TAAGTTAATTATTAAAACTTTCAACAATGGATCTCTTGGTTCTGGCATCGA

TGAAGAACGCAGCGAAATGCGATAAGTAGTGTGAATTGCAGAATTCAGTG

AATCATCGAATCTTTGAACGCACATTGCGCCCCTTGGTATTCCATGGGGCA

TGCCTGTTCGAGCGTCATTTGAACCCTCAAGCTCTGCTTGGTGTTGGGTGT

TTGTCCCGCCATTGCGCGCGGACTCGCCTTAAAGCAATTGGCAGCCATGT

AATCCGGCTTTGAGCGCAGCACATTGCGTACTCTCTACTGGGACTTGGCAT

CCAGAAGCCTTATTTTTTACTCTTGACCTCGGATCAGGTAGGGAACCCGCT

GAACTTAAGCTATCAATAAGCGGAGG

>P6 [organism=*Paecilomyces* sp.] [strain=P6]

TGTAATACACCTGTTGCTTCGGCGGGCCCGCCGTGGTTCACGCCGTGGCCG
CCGGGGGGCATCTCGCCCCCGGGCCCGCGCCCGCCGAAGACCCCTCGAAC
GCTGCCTTGAAGGTTGCCGTCTGAGTATGAAATTCAATCGTTAAAACTTTC
AACAACGGATCTCTTGGTTCCGGCATCGATGAAGAACGCAGCGAAATGCG
ATAAGTAATGTGAATTGCAGAATTCCGTGAATCATCGAATCTTTGAACGC
ACATTGCGCCCCCTGGCATTCCGGGGGGCATGCCTGTCCGAGCGTCATTG
CTAACCCTCCAGCCCGGCTGGTGTGTTGGGTCGACGTCCCCCCCGGGGGA
CGGGCCCGAAAGGCAGCGGCGGCGCCGCGTCCGATCCTCGAGCGTATGG
GGCTTTGTCACGCGCTCTGGTAGGGTCGGCCGGCTGGCCAGCCAGCGACC
TCACGGTCACCTATTATTTTTCTCTTAGGTTGACCTCGGATCAGGTAGGGA
TACCCGCTGATAAACT

>K2 [organism=*Penicillium* sp.] [strain=K2]

TAGTCCGGTGAACCTGCGGAAGGATCATTACCGAGTGAGGGCCCTCTGGG
TCCAACCTCCCACCCGTGTTTATTTTACCTTGTTGCTTCGGCGGGCCCGCCT
TAACTGGCCGCCGGGGGGCTCACGCCCCCGGGCCCGCGCCCGCCGAAGAC
ACCCTCGAACTCTGTCTGAAGATTGTAGTCTGAGTGAAAATATAAATTATT
TAAAACTTTCAACAACGGATCTCTTGGTTCCGGCATCGATGAAGAACGCA
GCGAAATGCGATACGTAATGTGAATTGCAAATTCAGTGAATCATCGAGTC
TTTGAACGCACATTGCGCCCCCTGGTATTCCGGGGGGCATGCCTGTCCGA
GCGTCATTACTGCCCTCAAGCACGGCTTGTGTGTTGGGCCCCGTCCTCCGA
TCCCGGGGGACGGGCCCGAAAGGCAGCGGCGGCACCGCGTCCGGTCCTC
GAGCGTATGGGGCTTTGTCACCCGCTCTGTAGGCCCGGCCGGCGCTTGCC

GATCAACCCAAACTTCTATAGGTTGACCTCGGATCAGGTAGGGATACCCG

CTGAACTTAAGCATATCAATAGGAGGC

>K3 [organism=*Penicillium* sp.] [strain=K3]

TAGTCCGGTGAACCTGCGGAAGGATCATTACCGAGTGAGGGCCCTCTGGG

TCCAACCTCCCACCCGTGTTTATTTACCTTGTTGCTTCGGCGGGCCCGCCT

TAACTGGCCGCCGGGGGGCTCACGCCCCCGGGCCCGCGCCCGCCGAAGAC

ACCCTCGAACTCTGTCTGAAGATTGTAGTCTGAGTGAAAATATAAATTATT

TAAAACTTTCAACAACGGATCTCTTGGTTCCGGCATCGATGAAGAACGCA

GCGAAATGCGATACGTAATGTGAATTGCAAATTCAGTGAATCATCGAGTC

TTTGAACGCACATTGCGCCCCCTGGTATTCCGGGGGGCATGCCTGTCCGA

GCGTCATTACTGCCCTCAAGCACGGCTTGTGTGTTGGGCCCCGTCCTCCGA

TCCCGGGGGACGGGCCCGAAAGGCAGCGGCGGCACCGCGTCCGGTCCTC

GAGCGTATGGGGCTTTGTCACCCGCTCTGTAGGCCCGGCCGGCGCTTGCC

GATCAACCCAAACTTCTATAGGTTGACCTCGGATCAGGTAGGGATACCCG

CTGAACTTAAGCATATCAATAGGAGGC

>N1 [organism=*Ceratobasidium* sp.] [strain= N1]

CCGATAGTGAACTTGCGGAAGACATTTATTTGAATGAATGTTCGAAGTTTG

GTTGTCCGCTTGGCCCTCTTGGGCATGTGCACGCCTTCTCTTTCATCCACA

CACACCTGTGCACTTGTGAGACGGAGGACTTTAATTAGTCTTCCGTCTACT

TAATTACACAAACTCATTTAATTAAATTGAATGTAATTGATGTAACGCATC

ATTAGAACTAAGTTTCAACAACGGATCTCTTGGCTCTCGCATCGATGAAG

AACGCAGCGAAATGCGATAAGTAATGTGAATTGCAGAATTCAGTGAATCA

TCGAATCTTTGAACGCACCTTGCGCTCCTTGGTATTCCTTGGAGCATGCCT

GTTTGAGTATCATGAAATTCTCAAAGTAAATCTTTTGTTAATTCAATTGGT

TTGCTTTGGACTTGGAGGTCTTTGCAGATTTCACGTCTGCTCCTCTTAAATG

CATTAGCTGGATCTCAGTATATGCTTGGTTCCACTCGGCGTGATAAGTATC

ACTCGCTGAGGACACCTTAAAAAGTGGCCAAGAAATACAGATGAACCGCT

TCTAATAGTCTATTAAGTTAGACAATTAATTTAAGATCTGATCTCAAATCA

GGTAGGACTACCCGCTGAACTTAAGCATACAA

>N2 [organism=*Ceratobasidium* sp.] [strain=N2]

ATGATGTAGAGTTGGTTGTCGCTGGCCCTCTTGGGCATGTGCACGCCTTCT

CTTTCATCCACACACACCTGTGCACTTGTGAGACGGAGGACTTTAATTATT

CTTCCGTCTACTTAATCACACAAACTCATTTAATTAAATTGAATGTAATTG

ATGTAACGCATCATTAGAACTAAGTTTCAACAACGGATCTCTTGGCTCTCG

CATCGATGAAGAACGCAGCGAAATGCGATAAGTAATGTGAATTGCAGAA

TTCAGTGAATCATCGAATCTTTGAACGCACCTTGCGCTCCTTGGTATTCCT

TGGAGCATGCCTGTTTGAGTATCATGAAATTCTCAAAGTAAATCTTTTGTT

AATTCAATTGGTTTGCTTTGGACTTGGAGGTCTTTGCAGATTTCACGTCTG

CTCCTCTTAAATGCATTAGCTGGATCTCAGTATATGCTTGGTTCCACTCGG

CGTGATAAGTATCACTCGCTGAGGACACCTTAAAAAGTGGCCAAGAAATA

CAGATGAACCGCTTCTAATAGTCTATTAAGTTAGACAATTAATTTAAGATC

TGATCTCAAATCAGGTAGGACTACCCGCTGAACTTAAGCATATCAATAAG

CGGAGGAAAAGAAACTAACAAGGATTCCCCTAGTAACGGCGAGTGAAGC

GGGA

> N3 [organism=*Ceratobasidium* sp.] [strain=N3]

GGAAGTAAAAGTCGTAACAAGGTTTCCGTAGGTGAACCTGCGGAAGGATC

ATTATTGAATGAATGTAGAGTTGGTTGTCGCTGGCCCTTTCGGGGGTATGT

GCACGCCTTCTCTTTCATCCACACACACCTGTGCACTTGTGAGACGGAGGG

CTTTAATTAGTCTTCCGTCTATTCAACCACACAAACTCATTGTATTTAAAC

TGAATGTAATTGATGTAACGCATCATTAGAACTAAGTTTCAACAACGGAT

CTCTTGGCTCTCGCATCGATGAAGAACGCAGCGAAATGCGATAAGTAATG

TGAATTGCAGAATTCAGTGAATCATCGAATCTTTGAACGCACCTTGCGCTC

CTTGGTATTCCTTGGAGCATGCCTGTTTGAGTATCATGAAATTCTCAAAGT

AAATCTTTTGTTAATTCAACTGGTTTTGCTTTGGACTTGGAGGTCTTTGCAG

ATTTCACGTCTGCTCCTCTTAAATGCATTAGCTGGATCTCAGTATATGCTT

GGTTCCACTCGGCGTGATAAGTATCACTCGCTGAGGACACTGTAAAAGGT

GGCCAGGAAATACAGATGAACCGCTTCTAATAGTCTATTAAGTTAGACAA

TTTAATTTTAAGATCTGATCTCAATCAGGTAGGACTACCCGCTGAACTTAA

GCATATCAGCGGATAA

>N4 [organism=*Ceratobasidium* sp.] [strain= N4]

CCGATAGTGAACTTGCGGAAGACATTTATTTGAATGAATGTTCGAAGTTTG

GTTGTCCGCTTGGCCCTCTTGGGCATGTGCACGCCTTCTCTTTCATCCACA

CACACCTGTGCACTTGTGAGACGGAGGACTTTAATTAGTCTTCCGTCTACT

TAATTACACAAACTCATTTAATTAAATTGAATGTAATTGATGTAACGCATC

ATTAGAACTAAGTTTCAACAACGGATCTCTTGGCTCTCGCATCGATGAAG

AACGCAGCGAAATGCGATAAGTAATGTGAATTGCAGAATTCAGTGAATCA

TCGAATCTTTGAACGCACCTTGCGCTCCTTGGTATTCCTTGGAGCATGCCT

GTTTGAGTATCATGAAATTCTCAAAGTAAATCTTTTGTTAATTCAATTGGT

TTGCTTTGGACTTGGAGGTCTTTGCAGATTTCACGTCTGCTCCTCTTAAATG

CATTAGCTGGATCTCAGTATATGCTTGGTTCCACTCGGCGTGATAAGTATC

ACTCGCTGAGGACACCTTAAAAAGTGGCCAAGAAATACAGATGAACCGCT

TCTAATAGTCTATTAAGTTAGACAATTAATTTAAGATCTGATCTCAAATCA

GGTAGGACTACCCGCTGAACTTAAGCATACAA

>N5 [organism=*Ceratobasidium* sp.] [strain= N5]

CCGATAGTGAACTTGCGGAAGACATTTATTTGAATGAATGTTCGAAGTTTG

GTTGTCCGCTTGGCCCTCTTGGGCATGTGCACGCCTTCTCTTTCATCCACA

CACACCTGTGCACTTGTGAGACGGAGGACTTTAATTAGTCTTCCGTCTACT

TAATTACACAAACTCATTTAATTAAATTGAATGTAATTGATGTAACGCATC

ATTAGAACTAAGTTTCAACAACGGATCTCTTGGCTCTCGCATCGATGAAG

AACGCAGCGAAATGCGATAAGTAATGTGAATTGCAGAATTCAGTGAATCA

TCGAATCTTTGAACGCACCTTGCGCTCCTTGGTATTCCTTGGAGCATGCCT

GTTTGAGTATCATGAAATTCTCAAAGTAAATCTTTTGTTAATTCAATTGGT

TTGCTTTGGACTTGGAGGTCTTTGCAGATTTCACGTCTGCTCCTCTTAAATG

CATTAGCTGGATCTCAGTATATGCTTGGTTCCACTCGGCGTGATAAGTATC

ACTCGCTGAGGACACCTTAAAAAGTGGCCAAGAAATACAGATGAACCGCT

TCTAATAGTCTATTAAGTTAGACAATTAATTTAAGATCTGATCTCAAATCA

GGTAGGACTACCCGCTGAACTTAAGCATACAA

>N6 [organism=*Ceratobasidium* sp.] [strain= N6]

CCGATAGTGAACTTGCGGAAGACATTTATTTGAATGAATGTTCGAAGTTTG

GTTGTCCGCTTGGCCCTCTTGGGCATGTGCACGCCTTCTCTTTCATCCACA

CACACCTGTGCACTTGTGAGACGGAGGACTTTAATTAGTCTTCCGTCTACT

TAATTACACAAACTCATTTAATTAAATTGAATGTAATTGATGTAACGCATC

ATTAGAACTAAGTTTCAACAACGGATCTCTTGGCTCTCGCATCGATGAAG

AACGCAGCGAAATGCGATAAGTAATGTGAATTGCAGAATTCAGTGAATCA

TCGAATCTTTGAACGCACCTTGCGCTCCTTGGTATTCCTTGGAGCATGCCT

GTTTGAGTATCATGAAATTCTCAAAGTAAATCTTTTGTTAATTCAATTGGT

TTGCTTTGGACTTGGAGGTCTTTGCAGATTTCACGTCTGCTCCTCTTAAATG

CATTAGCTGGATCTCAGTATATGCTTGGTTCCACTCGGCGTGATAAGTATC

ACTCGCTGAGGACACCTTAAAAAGTGGCCAAGAAATACAGATGAACCGCT

TCTAATAGTCTATTAAGTTAGACAATTAATTTAAGATCTGATCTCAAATCA

GGTAGGACTACCCGCTGAACTTAAGCATACAA
