# Supplementary material for: Diversity and antimicrobial activity of endophytic fungi isolated from Securinega suffruticosa in the Yellow River Delta
Source: PLoS One. 2020 Mar 10;15(3):e0229589. doi: 10.1371/journal.pone.0229589 (PMC7064225; doi:10.1371/journal.pone.0229589)
Supplement: Attachment — Submitted filename: Response to Reviewers.doc [file pone.0229589.s005.doc]

Dear Editor and Reviewers:

All authors agree to submit the work to PLOS ONE; the work has not been published and submitted to another journal. The order of author is Wen Du, Zhigang Yao, Jialiang Li, Chunlong Sun, Jiangbao Xi, Baogui Wan, Dongli Shi, Lili Ren. Wen Du is first author; Wen Du and Chunlong Sun are corresponding authors. Endophytic fungi in *Securinega* *suffruticosa* is the focus of research, so many researchers attempted to study it. I hope to expedite the review of my manuscript.

Thank you for your letter and for the reviewers’ comments concerning our manuscript entitled “Diversity and antimicrobial activity of endophytic fungi isolated from *Securinega suffruticosa* in the Yellow River Delta” (Manuscript Number: PONE-D-19-22808). Those comments are all valuable and very helpful for revising and improving our paper, as well as the important guiding significance to our researches. We have studied comments carefully and have made correction which we hope to meet with approval. The original article has been basically changed, and the modification is too large.

The following are responses to the reviewer's comments on revision.

**Responds to the editor’s comments:`**

1. Professional English proofreading of your manuscript is necessary before resubmission; Rewrite Introduction - paragraphs describing endophytes and Securinega suffruticosa - *the same beginning of each sentence, e.g.* Endophytic fungi and S. suffruticosa.;

**Response:** We rewrote the preface. The English translation company has revised it.

2. Shorten the title of your manuscript;

**Response:** The title changed from “Biodiversity and characterization of culturable endophytic fungi isolated from *Securinega suffruticosa* in Shell Islands of the Yellow River Delta, and analysis of their antimicrobial activity” to “Diversity and characterization of endophytic fungi isolated from *Securinega suffruticosa* in the Yellow River Delta, and analysis of their antimicrobial activity”.

Reviewer #1: Methodology:
a) The samples of *Securinega suffruticosa* were collected during two sampling years (2017 and 2018). It would be interesting to show the results of the culturable endophytic fungal composition between the two sampling periods.

**Response:** The another supplementary experiment was conducted to make the whole experiment more complete in 2018.

b) Secondly the protocol for endophyte isolation used in this study does not guarantee that all the culturable fungal diversity was obtained. I suggest that in addition to plating the 10 cm tissue sections on PDA agar, you could also crush the tissue samples using motor and pestle and perform serial dilutions. This will allow more surface area for the fungi to grow (slow growing fungi can be obtained efficiently) which may have not been isolated as the fast growing fungi dominates the incision/cut area of the 10 cm tissue.

**Response:** It has been cut into small sections of 10 cm before disinfection, and then cut into small pieces of 0.5cm × 0.5cm after disinfection. It is hoped that more endophytic fungi can still be isolated after removing the microorganisms on the surface.

Results:
a) As stated above, your results could also show the number of fungal isolates obtained between sampling periods.

**Response:** There was at first, but it was limited by the length of the article. Moreover, this part of data is only the increase of workload and lacks certain depth.

b) Statistical inferences (e.g. ANOVA) needs to be generated for the data on the dominance (Y) values of endophytic fungi in tissues, antagonism of endophytic fungal isolates, etc.
**Response:** When statistical calculation is needed, we try to add as much as possible, but it is not reasonable to add anova on the dominant value and antagonism.

Reviewer #2: In the manuscript presented for review, the authors present the results of experiments concerning the isolation and partial characterization of endophytic fungi from Securinega suffruticosa. The initial number of isolates was reduced to 57 for which ITS region sequences were obtained.
The description of the research makes it possible to repeat it, but I have a few questions about the methodology.

P7; L4-5 - Whether the strains came from public accessible collections of microorganisms and had identification numbers? Where they a type strains? What was the source of Fusarium oxysporum and Phoma herbarum?

**Response:** The test strains were provided by the Biopharmaceutical Center of Binzhou University and the institute of Biochemistry and Nutrition of Guizhou University. The test strains included *Fusarium oxysporum* and *Phoma herbarum.*

P8; L6-8 – What was the result of surface disinfection? Is it possible to avoid the fungal growth on PDA medium when the surface sterilised parts of plant contain endophytic fungi and their release from tissues is very probable? Why the water from the last rinse was not tested for fungal presence? How long was the control incubated?

**Response:** We hope to remove the microorganisms on the surface of the plant as much as possible, and the control was cultured for 7 days. If some endophytic fungi released by plant tissues are sterilized and removed, the endophytic fungi can also be present in plant tissues.

P11; L17-18 - What method was used to determine the number of bacteria in the inoculum?
**Response:** Take a small amount to measure the concentration through microscope, add sterile water to adjust the concentration.。

P12; L20 – Is it possible to obtain more specified than total alkaloid content data? Chromatographic data (e.g. securinice) are strongly recommended.

**Response:** We will study the metabolites of individual strain. This experiment is mainly about “Biodiversity and characterization of culturable endophytic fungi and analysis of their antimicrobial activity”. Research papers also have a theme.

Results:
P15; L7-8 vs. P14; L3-4 – In my opinion the results obtained from phylogenetic studies concern 57 isolates (57 ITS sequences were obtained, not 420). Morphological analysis does not allow for unambiguous classification of isolates.
**Response:** Morphological analysis does not provide a clear classification. So we did the molecular identification.

P18; L19-22 and P19; L1-8 - I suggest rebuilding the entire chapter because it is currently written in a chaotic manner

P19; L1 – Table 5
P19; L5 – the description does not correspond with data presented in Table 6, e.g. “the crude extracts of C. globosum and C. ramotenellum (1.00 mg/mL) were enough to inhibit S. aureus”. For C. ramotenellum the MIC/MBC values are 2.00/2.00.

**Response:** We reorganized the chapter, and there were some problems with irregularities.

We tried our best to improve the manuscript and made some changes in the manuscript. We appreciate for Editors/Reviewers’ warm work earnestly, and hope that the correction will meet with approval.

Once again, thank you very much for your comments and suggestions.
